# Residual Feed Intake as an Efficiency Metric for Pre-Weaning Dairy Calves: What Do We Know?

**DOI:** 10.3390/life13081727

**Published:** 2023-08-11

**Authors:** Camila S. da Silva, Juliana M. Leão, Camila F. A. Lage, Sandra G. Coelho, Mariana M. Campos

**Affiliations:** 1 Brazilian Agricultural Research Corporation, Embrapa Dairy Cattle, Juiz de Fora 36038-330, Minas Gerais, Brazil; camilaszootecnia@gmail.com; 2 The Saskatoon Colostrum Company, Ltd., Saskatoon, SK S7K 6A2, Canada; juliana.merghleao@sccl.com; 3 Cornell Cooperative Extension, Ithaca, NY 14850-3555, USA; cd546@cornell.edu; 4 Department of Animal Science, School of Veterinary Medicine, Federal University of Minas Gerais, Belo Horizonte 30161-970, Minas Gerais, Brazil; sandragesteiracoelho@gmail.com

**Keywords:** efficiency testing, high efficiency, low efficiency, young cattle

## Abstract

Dairy cattle systems have targeted improvements in feed efficiency by selecting animals that can convert less feed into more products. Residual feed intake (RFI) has been the index of choice when selecting dairy cattle for feed efficiency. Nonetheless, RFI studies have focused on lactating cows, and the crucial importance of pre-weaning efficiency on farm profitability and cow productivity has been mostly neglected. This review discusses the current knowledge of how RFI divergence relates to nutrient metabolism in pre-weaning dairy calves, including the advantages and limitations of evaluating RFI in this phase. Existing literature indicates that nutrient utilization, energy metabolism, protein metabolism, vitamin metabolism, intestinal development, and hindgut bacterial populations may be implicated in RFI divergence between pre-weaning calves. Techniques developed to date to evaluate RFI in this phase are still evolving to better adapt to the unique characteristics of this phase, and more research is needed to fill in the gaps in our current understanding of early-life feed efficiency divergence in cattle. However, current results suggest great potential for selecting high-efficiency calves while in pre-weaning to accelerate the progress of genetic selection in dairy cattle.

## 1. Introduction

Feed expenses have accounted for 30 to 70% of the total costs of milk production over the past two decades [1]. Hence, improving feed utilization efficiency has always been of great interest in dairy farms and has gained even more attention with the pressing environmental concerns about the impact of livestock on greenhouse gas emissions [2].

Ruminants have been intensively selected for a combination of feed efficiency and milk production traits. Residual feed intake (RFI) has been included in genetic selection programs in some countries [3,4] as a measure of feed efficiency in dairy cattle production systems. RFI was originally proposed in 1963 [5] and can be defined as the difference between observed and predicted intake. The residual is generated by a multiple regression model where dry matter intake (DMI) is regressed on average daily gain (ADG) and BW^0.75^ [6]. Negative RFI values refer to the most efficient animals during the testing period and exhibit a lower feed intake than predicted for a given weight gain.

Most RFI studies in dairy cattle have focused on lactating cows. Because in lactation, orchestrated adaptations prioritize a certain physiological state, estimates of RFI in lactating cows need to account for additional sources of variation aside from ADG and BW^0.75^, such as body maintenance, possible pregnancy, milk production, parity, and stage of lactation [7].

Some studies observed a repeatability of RFI from growing to lactating phase [8,9], indicating that RFI may be a lifetime trait. Thus, the determination of RFI in young calves may be simpler and cheaper and might accelerate genetic progress [10]. Most published studies focus on weaned calves, despite the impact of pre-weaning on the productive success of future cows and on feeding expenses in dairy farms [11]. Hence, the objective of this review is to summarize the current knowledge of feed efficiency in pre-weaned dairy calves, focusing on the application of RFI as the feed efficiency measure, and discuss the potential and future research directions in this area.

## 2. Feed Efficiency in the Pre-Weaning Phase

The energy expenditure for body maintenance is a key component in feed efficiency variations among different animals [12]. Maintenance requirements refer to the amount of energy required for basal metabolism, such as protein synthesis and degradation, ion transport, cell signaling, functioning of vital organs, voluntary movements, and thermoregulation [13]. The net energy required for maintenance has been recently estimated at 70.2 kcal/metabolic body weight (BW^0.75^) per day for pre-weaning Holstein and crossbred Holstein × Gyr calves, with an efficiency of utilization of metabolizable energy for maintenance at 66% [14]. It has been demonstrated that high-efficiency cattle spend less energy on physiological processes related to body maintenance, sparing more energy for tissue deposition [15]. However, only a few studies have attempted to identify the mechanism underlying RFI divergence in pre-weaning calves (Table 1).

RFI divergence in pre-weaning dairy calves was first investigated in 2018 [19] using Holstein × Gyr F1 crossbred calves. High- and low-efficiency calves presented RFI values of −0.14 g/day and 0.13 g/day. In addition, calves grouped as low RFI were 13% more efficient than animals classified as high RFI. RFI significantly influenced dry matter intake, gas exchanges, and heat production in this study, and subsequent research [18] also showed differences in nutrient digestibility, nitrogen metabolism, and rumen function between RFI-divergent Gyr pre-weaning calves. Unlike measures of metabolic function, RFI appears to be unrelated to body measurements in the pre-weaning phase [16,17,20], indicating that selection for RFI at early ages does not affect the growth or body size of adult animals [21].

## 3. Relationship between RFI and Nutrient Metabolism in Pre-Weaning Dairy Calves

Albeit tissue catabolism and anabolism, activity, and thermoregulation play a significant role in feed efficiency divergence in cattle [22], feed efficiency divergence is generally attributed to differences in nutrient intake and digestion. The relationship between RFI and several variables related to feed intake and nutrient utilization in pre-weaning calves are discussed below and have been summarized in Table 2.

### 3.1. Effects of RFI on Heat Increment and Gas Exchanges

Feed intake is mainly driven by maintenance requirements; hence, the amount of energy spent on feed digestion, or simply heat increment, is proportional to feed intake. Thus, heat increment increases as feed intake increases [22]. It has been estimated that heat increment can account for 75% of total energy intake [23]. Consequently, animals that can consume less feed but convert more gross energy into net energy are considered more efficient.

Traditionally, heat increment has been estimated through indirect calorimetry, which measures oxygen (O_2_) uptake and carbon dioxide (CO_2_) and methane (CH_4_) production by animals using open-circuit respiration chambers [24,25]. An alternative method, called “the face mask method”, has also been proposed [26]. In this method, energy expenditure is estimated based on gas exchanges but, unlike the chambers, measured using a face mask. Because O_2_ uptake is directly related to feed intake [27], it has been suggested that O_2_ uptake is lowest in efficient animals. Indeed, RFI was positively correlated with heat production (r = 0.48), O_2_ uptake (r = 0.48), CO_2_ production (r = 0.48), and heart rate (r = 0.40) in pre-weaning dairy calves [19]. In addition, high-efficiency calves spent 15.3% less energy as heat, exhibited a lower heart rate, consumed less O_2_, and produced less CO_2_ compared to high-RFI animals.

Data collected in that trial [19] were acquired using face masks. When gas exchanges were evaluated via respirometric chambers, no differences in O_2_ uptake or CO_2_ and heat production between low and high-efficiency calves were observed [17]. The discrepancy in these results may be related to the difference in feed intake between the low- and high-efficiency groups reported in the two studies: in the former, calves in the high-efficiency group consumed 320 g less milk + starter than low-efficiency calves, whereas the latter study reported that the difference in feed intake between the two groups was only 80 g and mainly driven by lower starter intake.

Notwithstanding statistical significance, this difference might not have been large enough to cause detectable changes in gas production and heat increment. Furthermore, data behavior acquired from 1049 dairy heifers housed in outdoor facilities and classified as low or high efficiency at 5–9 months of age have shown differences in feeding behavior over 24 h, with the most efficient animals eating less and having fewer meals during daylight (0600 to 2100 h), especially during the afternoon (1200 to 1800 h), but eating for a longer time during the night (0000–0600 h) than the least-efficient animals [28]. Nonetheless, behavior patterns might not reflect the regular calf behavior for animals placed inside respirometric chambers, and the potential effects of the chamber’s environment on feed intake and energy utilization cannot be ruled out [29].

### 3.2. Effects of RFI on Nutrient Digestibility

In pre-weaning dairy calves, it has been suggested that differences in feed utilization between RFI-divergent groups may be related to greater intestinal activity due to the large contribution of intestinal digestion [17]. In broad terms, nutrient digestibility is expected to be lower in the least efficient cattle because of the higher feed intake [30]. High-efficiency pre-weaning calves (RFI = −0.052 kg/day) had greater digestibility of crude protein and ether extract and tended to show a higher total dry matter and organic matter digestibility compared to low-efficiency (RFI = 0.049 kg/day) calves [17].

Small intestine mass (g) and relative small intestinal mass (g/kg BW) were positively correlated with dry matter intake and RFI in young cattle [31], and high efficiency was negatively correlated with crypt perimeter, crypt area, and nuclei number in duodenum cells [32]. Tissues of the splanchnic bed, which include the gastrointestinal tract, liver, spleen, pancreas, mesenteric fat depots, connective tissue, and blood vessels, comprise 15 to 20% of the total body mass in ruminants [33], and O_2_ uptake by the portal-drained viscera can reach 25% [34]. These observations suggest that efficient animals have a lower energy requirement for tissue maintenance as well as a greater ability to acquire nutrients per unit of small intestinal mass [31]. In fact, low efficiency was positively correlated (r = 0.50) with a greater gross energy intake in pre-weaning calves [17].

### 3.3. Effects of RFI on Nitrogen (N) Metabolism

The efficiency of N utilization in ruminants is typically low due to the several steps involved in peptide degradation in these animals [35]. The introduction of RFI in selection programs increased the interest in exploring how RFI divergence affects nitrogen metabolism [36]. However, studies revealing the potential effects of divergent RFI on N utilization in pre-weaning dairy calves are almost inexistent.

RFI was moderately and positively correlated with nitrogen intake (r = 0.50) and fecal losses (r = 0.60) in pre-weaning calves [17], supporting the observation of lowest CP digestibility by low-efficiency animals. Moreover, low-efficiency calves tended to present higher blood urea levels (21.4 mg/dL) than high-efficiency calves (18.4 mg/dL) [18]. Considering that urea is a product of protein degradation, this could be a result of protein catabolism [18] or lower N recycling in the least efficient animals, as suggested for lactating cows. Nonetheless, the lack of differences in urinary N and N retention between low- and high-efficiency calves indicates that more research is needed to elucidate the N metabolism in this rearing phase.

Recent results obtained with lactating cows show an association between RFI and milk protein efficiency, expressed as dietary protein captured in milk [37]. Cows classified as most efficient based on RFI utilize dietary N for milk protein and body N synthesis more efficiently [38]. Possible reasons for improved efficiency of N utilization in most efficient lactating cows may include greater N digestibility and N recycling to the gut [38], which has also been suggested for pre-weaning calves [17] and 90-day-old lambs [36].

### 3.4. Effects of RFI on Rumen Fermentation Profile

Changes in rumen fermentation products have been reported in pre-weaning calves classified as low or high efficiency [18] from 28 to 56 days of age; low-efficiency calves had lower molar concentrations of VFAs and propionate (% VFAs) and a greater proportion of acetate in rumen fluid compared to high-efficiency animals. However, these differences were not linked to RFI per se but rather to the higher intake of starter feed by low-efficiency calves, which may have increased the passage rate and altered the ratios of absorption and digestion. In addition, no differences in pH or ammonia concentration were found, suggesting similar fermentation conditions between RFI groups. Subsequent results [20] also reported no effects of RFI divergence on the fermentation profile of pre-weaning calves, and rumen fermentation variables were not correlated with RFI.

The lack of correlation between rumen fermentation parameters and RFI in the pre-weaning phase is probably related to insufficient rumen function in milk-fed animals, as neonatal calves have an underdeveloped rumen until close to weaning [16], and differences in rumen fermentation products have been reported for RFI-divergent animals in later life [39,40]. In fact, β-hydroxybutyrate levels have been pointed out as a potential marker for the identification of high-efficiency heifers post-weaning [40]. It is possible that gene expression in rumen epithelium [41] and epithelium-associated bacteria [42], rather than fermentation, might be involved in improved efficiency in pre-weaning calves, as it has been indicated for older cattle.

### 3.5. Effects of RFI on Hindgut Microbiome

In lactating cows, low and high efficiency of milk production has been correlated with variations in the abundances of certain rumen bacterial communities [43]. Because pre-weaning digestion is marked by substantial hindgut fermentation of undigested diet components, it has been suggested that the hindgut microbiome is implicated in RFI divergence between young calves. Indeed, Elolimy et al. [16] have demonstrated that RFI is associated with unique hindgut microbiome and metabolome profiles in neonatal Holstein heifer calves. Furthermore, they observed a maternal and pre-weaning nutrition effect of hindgut microbial communities and metabolome profile.

High-efficient calves had a lower abundance of pathogenic bacteria at birth, such as *Odoribacter*, *Cyanobacteria, Ruminiclostridium* 9, *Prevotellaceae* UCG-001, and *Eubacterium nodatum*, indicating superior hindgut functionally in these animals. In addition, high-efficient calves had a greater number of functional genes involved in VFA biosynthesis. The analysis of the hindgut metabolome also showed significant differences in the metabolic profile between low- and high-efficiency calves at birth. High-efficient calves had greater enrichment of key metabolites involved in energy-generating pathways, including the citric acid cycle, gluconeogenesis, and pyruvate metabolism, potentially enhancing the supply of energy to the calf and the ability to activate metabolic pathways for amino acid, vitamin, and fatty acid metabolism, which could benefit hindgut development and function [16]. On the contrary, high-efficient calves downregulated metabolites associated with the inhibition of several pathways, such as folate metabolism, amino sugar metabolism, sphingolipid metabolism, steroidogenesis, and bile acid biosynthesis.

Interestingly, microbiome communities shifted in response to RFI divergence during the pre-weaning period [16]. The results revealed an increased abundance of several carbohydrate-fermenting bacteria in high-efficiency calves, suggesting a greater capacity for utilizing complex carbohydrates reaching the hindgut and improved colonocyte growth and function through the production of VFAs [44].

The microbiome of low- and high-efficiency calves also exhibited distinct vitamin and amino acid metabolism [16]. Synthesis of vitamin B7, vitamin B6, and vitamin B9, as well as the amino acids arginine, proline, methionine, tyrosine, tryptophan, and phenylalanine, is upregulated in high-efficiency calves, suggesting a positive association between high efficiency and reduced oxidant status and a line of communication between hindgut and brain during the pre-weaning period. Catabolism of branched-chain amino acids (BCAA) was also upregulated in the high-efficient calves. Degradation of BCAA generates α-keto acids, known for promoting cellular growth through the activation of the mechanistic target of rapamycin (mTOR) signaling [45]. As with neonatal calves, steroid, and bile acid synthesis was downregulated in high-efficiency calves during pre-weaning.

A study investigating mucosa and digesta bacterial communities throughout the gastrointestinal tract (GIT) of pre-weaned calves [46] has identified that the prevalence of *Prevotella*, *Bacteroides*, *Lactobacillus*, and *Faecalibacterium*, which were among selected abundant bacteria, was significantly different among the GIT regions and between mucosa- and digesta-associated communities. The rumen contained the most diverse bacterial population, comprising 47 genera in total and 16 rumen-specific genera. Additionally, it has been acknowledged that bovine ruminal bacterial populations change from birth to adulthood [47]. It is possible that rumen bacterial communities and colonization are associated with RFI divergence among pre-weaning calves. Future studies exploring the connection between RFI and rumen bacterial diversity colonization could deepen our understanding of feed efficiency in early life and whether pre-weaning rumen bacterial diversity is linked to RFI during lactation.

## 4. Advantages and Limitations for Feed Efficiency Evaluations in Pre-Weaning Calves

**Figure 1:**
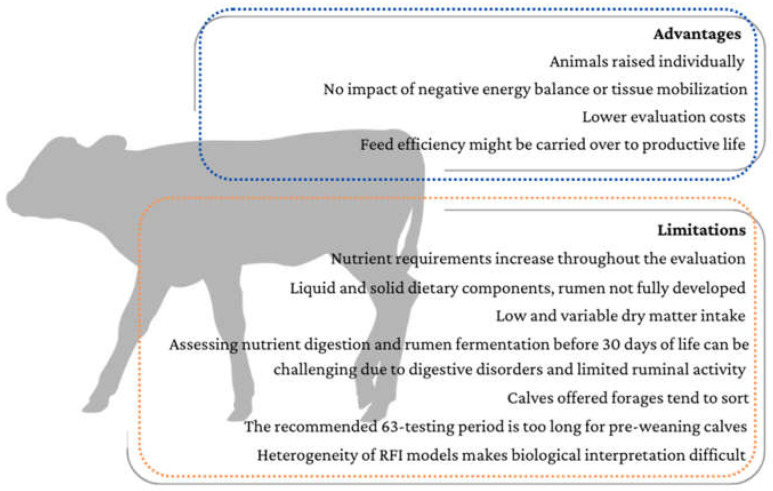
Summary of main advantages and limitations of measuring calf RFI in the pre-weaning phase.

As previously mentioned, the advantages of assessing RFI in the pre-weaning phase lie in that pre-weaning calves are raised individually, facilitating individual measurements of feed intake. Moreover, pre-weaning RFI models are not subjected to the effects of negative energy balance, pregnancy, or tissue mobilization, evaluation costs may be lower compared to post-weaning or lactating phases, and the repeatability of RFI may accelerate improvements of the herd feed efficiency through genetic selection of young calves. On the other hand, the particularities of feeding management and nutrient digestion in the pre-weaning phase impose limitations on methods developed to date for assessing RFI and its relationship with nutrient metabolism in pre-weaning calves (Figure 1). These include:

- The growth requirements of dairy calves change daily during the evaluation period. However, in commercial settings, calves receive fixed amounts of milk or replacer throughout the pre-weaning phase, regardless of their body weight at birth. This practice gives lighter calves an advantage, as they receive more nutrients from the liquid diet. Ideally, milk offer should be adjusted according to the calves’ metabolic weight and increased as they grow. However, this is not feasible in classic commercial settings. The use of automated milking feeders during RFI evaluations in pre-weaning calves can help overcome these issues, as different liquid diet allowances can be provided over time.
- Pre-weaning calves receive nutrients in both liquid and solid form, and digestion occurs in different parts of their digestive system, mainly the abomasum/intestine and developing rumen. Liquid and solid diets have different digestibilities, with liquid diets usually being more digestible. Therefore, the individual proportions of liquid and solid intake can introduce bias in RFI evaluations. Developing equations to account for this can potentially improve the accuracy of the evaluations during this phase.
- Since overall intake during the pre-weaning phase is low and variable, it can be challenging to detect differences in dry matter intake and other variables between RFI groups. Increasing the number of animals enrolled in efficiency trials can improve the accuracy of the evaluations.
- Assessing nutrient digestion before 30 days of age can be challenging due to the low intake of a solid diet and the higher incidence of digestive disorders and diseases in the first two weeks of life. In addition, digestive disorders in the first two weeks of life usually depress feed intake and add additional bias to feed intake evaluations and digestibility values. Digestibility assays have a higher chance of success when performed in animals that are 35 days of age or older.
- Variations in rumen fermentation are also difficult to observe in the first 30 days of life due to limited ruminal activity.
- Using a homogenous concentrate for solid diets is preferred over using sortable concentrates. Sorting can affect the nutrient composition of the diet and introduce bias. If forage sources are offered, providing them separately from the concentrate may be ideal, as calves offered forage and concentrate together tend to sort, leading to differences in intake composition between RFI groups.
- The recommended 63-day observation period for RFI evaluations is lengthy for pre-weaning calves, considering that calves are usually on a milk diet for 60 days on commercial farms. The use of technology that allows for automatic daily measurements of body weights, body composition, and accurate values for liquid and solid intakes has the potential to provide consistent measurements and reduce the time needed to observe divergence in RFI for pre-weaning calves.
- As mentioned in previous studies [30,48], there is a complex array of results, correlations, and heritabilities in RFI studies, which often limit biological interpretations.

## 5. General Recommendations for Assessing RFI in Pre-Weaning Calves

To our knowledge, no specific protocols for RFI evaluations in dairy cattle have been established. Research investigating RFI in dairy cattle has adjusted to general recommendations for RFI assessment in beef cattle, which are based on studies published by Archer et al. and Wang et al. in 1997 and 2006, respectively, with growing beef cattle [49,50].

The Australian Standard Protocol for RFI testing in beef cattle [51] advises that animals must be fed a constant ration for at least 70 days following a 21-day adaptation period to obtain an accurate measure of RFI. The proposed 70-day test period seems the most indicated for animals weighed every two weeks [49]. However, Wang et al. indicated that 63 days should be sufficient to obtain an accurate measure of RFI if BW is measured weekly. Under these circumstances, relative changes in phenotypic residual variance become very small after 63 d of testing. Therefore, the testing period can be reduced with more frequent weighing.

The lack of specific protocols for evaluating RFI during pre-weaning is probably one of the main reasons why there is substantial literature on post-weaning and lactating phases but not on milk-fed calves. More importantly, the unique features of this phase require several adaptations to general methodologies described in studies carried out with growing and adult cattle. Based on the practical application of RFI methodologies to pre-weaning studies, we suggest the following recommendations for future evaluations of RFI in pre-weaning calves: enrolled calves should be of similar sex and breed/genetic composition; calves should be evaluated for at least 56 days; calves should be enrolled in the test after 14 days of life when intake of the solid feed has become more significant, and scours interference on feed intake and BW gain has been reduced; the solid feed should be highly homogeneous to avoid diet selection; calving dates should be as concentrated as possible to minimize age variations among contemporary calves and keep it under 60 days; due to the age differences, pre-established testing periods should be applied to calves individually. In that case, BW measurements should be taken based on the age of individual calves until the total testing period is complete; BW should be measured at least once a week to allow for proper adaptation to the test and reduction of the total test period; BW should be always be recorded around the same time, prior to the morning milk feeding; available facilities should allow individual assessment of dry matter intake and the distinction between intake of milk and the solid feed; milk offer should be adjusted for metabolic BW throughout the evaluation period.

## 6. Future Directions

Available techniques are still being adjusted to be more suitable for evaluating RFI in the pre-weaning phase. Further studies could benefit from refined technologies that will increase the accuracy of RFI evaluations. Future research is warranted to explore the roles of different nutritional strategies, nitrogen metabolism, rumen epithelium and microbiota, and digestion sites in RFI-divergent pre-weaning calves and clarify the mechanisms distinguishing efficient animals in this phase. Physiological differences between suckling calves, bucket-fed calves, and calves fed through automatic milk feeders should also be explored. Due to inconsistent results regarding the repeatability of RFI classification over phases, additional work should also focus on investigating the genetic repeatability of RFI from pre-weaning to productive life, shedding light on whether calves selected for high efficiency in the pre-weaning phase will remain most efficient in the growing and lactating phases and, therefore, be suitable for genetic selection of this trait.

## Figures and Tables

**Table 1:** Description of experimental conditions established for RFI testing in pre-weaning dairy calves.

Reference	n	Breed	Testing Period (Days)	Age at Test Enrollment (Days)	Age at Test Completion (Days)	Milk/Milk Replacer (kg/d)	Composition of Solid Diet	Weighing Frequency
[16]	26	Holstein (H)	42	1	42	5.3 ^1^	100% starter	weekly
[17,18]	32	Gyr (G)	63	14	77	4.7 ^2^	92% starter 8% Tifton hay	weekly
[19,20]	36	Girolando	56	25	80	6.0	95% starter 5% Tifton hay	weekly

^1^ Average intake of milk replacer obtained from the amount of milk replacer offered from days 1–10 (4.5 kg/d), days 11–20 (5.9 kg/d), days 21–35 (7.3 kg/d), and days 36–42 (3.6 kg/d). ^2^ A total of 42% of metabolic birth weight (4.7 ± 0.46).

**Table 2:** Effects of RFI divergence on feed efficiency, nutrient metabolism, and microbiome of pre-weaning calves.

Item	Variable	Low Efficiency	High Efficiency	Reference
Intake	RFI (kg/d)	0.130	−0.140 **	[19]
		0.049	−0.052 **	[17]
		0.168	−0.173 **	[16]
Performance	Average daily gain (kg/d)	0.98	0.98	[19]
		0.59	0.60	[17]
		0.57	0.56	[16]
Heat production	Heat production (kcal/d per BW^0.75^)	172	148 **	[19]
		628	586	[17]
Gas exchanges	O_2_ uptake (L/d)	787	668 **	[19]
		568	567	[17]
	CO_2_ production (L/d)	702	592 **	[19]
		525	534	[17]
Nutrient digestibility	Dry matter (%)	85.8	89.2 *	[17]
	Crude protein (%)	87.8	91.8 **	
	Ether extract (%)	93.1	96.4 **	
N metabolism	N intake (g/d/BW0.75)	1.74	1.56 **	[17]
	Urine N (g/d/BW0.75)	0.46	0.54	
	Fecal N (g/d/BW0.75)	0.21	0.13 **	
	Retained N	0.97	0.99	
Fermentation profile	pH	6.31	6.52	[18]
	Total VFAs (μmol/mL)	43.4	32.9	
	Acetate (% total VFAs)	0.67	0.75 **	
	Propionate (% total VFAs)	0.27	0.21 **	
Metabolomics	Energy-generating metabolites	-	Upregulated	[16]
i. Birth	Amino acid metabolism	-	Upregulated	
	*Odoribacter* ^1^	0.668	0.130 **	
	*Prevotellaceae* UCG-004 ^1^	0.233	0.149 **	
	*Ruminiclostridium* 9 ^1^	0.251	0.064 **	
ii. Pre-weaning period	*Fusobacterium* ^2^	0.167	0.230 **	
	*Succinivibrio* ^2^	0.012	0.020 **	
	*Bacteroides* ^2^	0.052	0.075 **	
	Vitamin B supply	-	Upregulated	
	Amino acid supply	-	Upregulated	
	BCAA ^3^ catabolism	-	Upregulated	

^1^ Pathogenic bacteria. ^2^ Carbohydrate-fermenting bacteria. ^3^ Branched-chain amino acids. * Significant at *p* ≤ 0.10. ** Significant at *p* ≤ 0.05.

## Data Availability

Not applicable.
